# GDF15 promotes glioma stem cell-like phenotype via regulation of ERK1/2–c-Fos–LIF signaling

**DOI:** 10.1038/s41420-020-00395-8

**Published:** 2021-01-11

**Authors:** Shan Zhu, Ning Yang, Yi Guan, Xue Wang, Guoxia Zang, Xinping Lv, Shuanglin Deng, Wan Wang, Tete Li, Jingtao Chen

**Affiliations:** 1 Institute of Translational Medicine, The First Hospital of Jilin University, Changchun, China; 2 Department of Neurosurgery, The First Hospital of Jilin University, Changchun, China

**Keywords:** Cell signalling, Cancer stem cells

## Abstract

Growth differentiation factor 15 (GDF15), a member of the transforming growth factor β family, is associated with tumor progression, metastasis, and cell apoptosis. However, controversy persists regarding the role of GDF15 in different tumor types, and its function in glioma stem cells (GSCs) remains unknown. Here, we report that GDF15 promotes the GSC-like phenotype in GSC-like cells (GSCLCs) through the activation of leukemia inhibitor factor (LIF)–STAT3 signaling. Mechanistically, GDF15 was found to upregulate expression of the transcription factor c-Fos, which binds to the LIF promoter, leading to enhanced transcription of LIF in GSCLCs. Furthermore, GDF15 may activate the ERK1/2 signaling pathway in GSCLCs, and the upregulation of LIF expression and the GSC-like phenotype was dependent on ERK1/2 signaling. In addition, the small immunomodulator imiquimod induced GDF15 expression, which in turn activated the LIF–STAT3 pathway and subsequently promoted the GSC-like phenotype in GSCLCs. Thus, our results demonstrate that GDF15 can act as a proliferative and pro-stemness factor for GSCs, and therefore, it may represent a potential therapeutic target in glioma treatment.

## Introduction

Glioma stem cells (GSCs) are a quiescent stem-like subpopulation of glioma cells with self-renewal and multi-lineage differentiation capacity^1^. GSCs are known to be resistant to chemotherapy^2^ and radiotherapy^3^. These cells are characterized by their expression of certain cancer stem cell markers, including CD133^4^, SOX2^5^, aldehyde dehydrogenase 1 family member A1 (ALDH1A1)^6^, and Nestin^7^. Under specific serum-free conditions in vitro, glioma cells form non-adherent spheroids known as neurospheres or tumorspheres (TS). The TS cells show GSC-like characteristics, including self-renewal, proliferation, and differentiation capacities^8^. GSCs are responsible for glioma initiation^9^; thus, they pose a great challenge to glioma therapy^10^. Hence, the identification of pivotal regulators of glioma stemness will provide critical information to advance the development of more effective glioma therapies.

Growth differentiation factor 15 (GDF15) acts as a tumor suppressor in the early stages of tumor development^11–13^, but later enhances the growth of high-grade tumors^14,15^. In glioma, GDF15 is involved in regulating cell proliferation and immune escape^16^; moreover, it has been correlated with poor patient prognosis and considered an oncogenic factor^17^. In solid tumors, GDF15 promotes the self-renewal capacity of cancer stem cells in gastric^18^, breast^19^, liver^20^, and ovarian cancers^21^. However, the function of GDF15 in GSCs remains to be elucidated.

Leukemia inhibitor factor (LIF), an interleukin- 6 (IL-6) family member, promotes the self-renewal of neural stem cells^22^ and GSCs^23,24^, and studies have even shown that recombinant LIF can be used as a serum-free medium supplement to increase the quantity of GSCs^4,25^. LIF binds to its receptor LIF-R and glycoprotein 130, and this binding event subsequently activates the JAK–STAT3 signaling pathway^26,27^. In GSCs, LIF is transcriptionally upregulated by transforming growth factor-beta (TGF-β) via binding of the activated Smad complex to the LIF promoter^23^. Since GDF15 is a member of the TGF-β family, we hypothesized that GDF15 may contribute to the regulation of glioma cell stemness via modulation of LIF–STAT3 signaling.

Here, we examined the role of GDF15 in GSC stemness and investigated the underlying molecular mechanism. Our work demonstrates that GDF15 upregulates LIF expression in GSC-like cells (GSCLCs) and promotes the GSC-like phenotype via the LIF–STAT3 pathway. Using transcriptome sequencing, we found that GDF15 significantly upregulates the transcription factor c-Fos, which binds to the LIF promoter responsible for its transcription. Moreover, the induction of LIF expression by GDF15 in GSCLCs is dependent on ERK1/2 signaling. Hence, our findings show a pro-tumor effect of GDF15 resulting from its promotion of GSC stemness.

## Results

### GDF15 promotes GSC-like phenotype in GSCLCs via activation of the LIF–STAT3 pathway

**Fig. 1:**
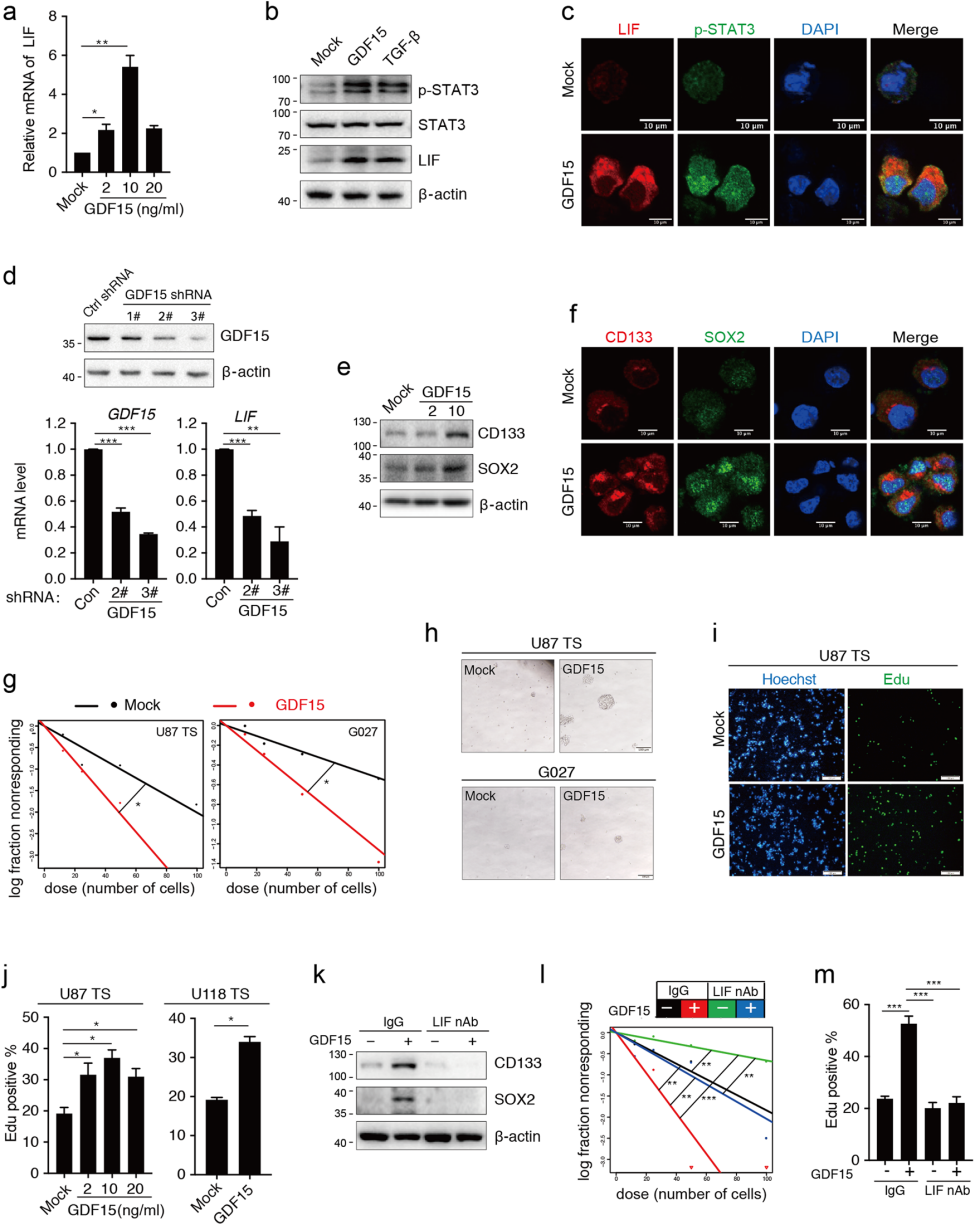
GDF15 induces the LIF–STAT3 pathway and enhances glioma stem cell (GSC)-like phenotype in GSC-like cells. **a** qRT-PCR analysis of LIF expression in U87 TS cells treated with 0, 2, 10, or 20 ng/ml GDF15. **b** Immunoblotting analysis of the levels of p-STAT3, STAT3, LIF, and β-actin in U87 TS cells after treatment with 10 ng/ml GDF15 and 100 pM TGF-β. **c** Immunocytochemical analysis with anti-LIF and anti-p-STAT3 in U87 TS cells. Nuclei were counterstained with DAPI. Scale bar = 10 μm. **d**, upper: Immunoblotting analysis of GDF15 expression after treatment of U87 TS cells with shRNA lentivirus targeting the GDF15 gene and control lentivirus. Lower: qRT-PCR analysis of GDF15 and LIF expression in U87 TS cells with short hairpin RNA lentivirus-mediated knockdown of GDF15. **e** Immunoblotting analysis of CD133, SOX2, and β-actin expression in U87 TS cells after treatment with GDF15 for 5 days. **f** Immunocytochemical analysis with anti-CD133 and anti-SOX2 in U87 TS cells. Nuclei were counterstained with DAPI. Scale bar = 10 μm. **g** Effects of GDF15 on the sphere-forming capacity of U87 TS and G027 cells as determined by extreme limiting dilution assay (*n* = 12). *P < 0.05; likelihood ratio test. **h** Representative images of U87 TS and G027 cells treated with 10 ng/ml GDF15 for 10 days. Scale bar = 100 μm. **i**, **j** EdU incorporation in U87 TS cells treated with GDF15 for 4 days, followed by EdU for 24 h, and quantification of EdU-positive cells. Nuclei were counterstained with Hoechst stain. Scale bar = 100 μm. **k** Immunoblotting analysis of CD133, SOX2, and β-actin expression in U87 TS cells after treatment with GDF15 and LIF-neutralizing antibodies (nAb) for 5 days. **l** The effects of LIF neutralization on the GDF15-induced sphere-forming frequency of U87 TS cells based on extreme limiting dilution assay (*n* = 12). **P < 0.005, ***P < 0.0005; likelihood ratio test. **m** Cell division was determined by the EdU incorporation assay in U87 TS cells treated with GDF15 and anti-LIF-neutralizing antibodies. Values in **a**, **d**, **j**, and **m** are from three independent experiments and expressed as mean ± s.e.m. *P < 0.05, **P < 0.005, ***P < 0.0005. Unpaired *t* test (**a**, **d**, **j**); one-way ANOVA with Tukey’s multiple comparison test (**m**).

The activation of the LIF–STAT3 signaling pathway by TGF-β in GSCs was previously reported^23^. Thus, we investigated whether GDF15, a member of the TGF-β superfamily, can regulate LIF expression, promoting the stemness of GSCs. To analyze the impact of GDF15 on GSCs, we established patient-derived and glioma cell line-derived TS cells (Supplementary Fig. 1), and the cells with GSC-like characteristics were utilized as GSCLCs in studies of glioma stemness in vitro. As expected, upregulated levels of LIF and phosphorylated STAT3 (p-STAT3) were observed in the GDF15-treated GSCLCs (Fig. 1a–c). Downregulation of GDF15 was achieved by shRNA treatment and resulted in decreased expression of LIF (Fig. 1d). These results imply that the LIF–STAT3 pathway in GSCs may be regulated by GDF15.

To assess the impact of GDF15 on the maintenance of stemness, immunoblotting and extreme limiting dilution assay (ELDA) were applied to test GSC marker expression and the self-renewal ability in GSCLCs. Indeed, the GSC markers CD133 and SOX2 were expressed at higher levels in GDF15-treated U87 TS cells than their control counterparts (Fig. 1e, f). Further, recombinant GDF15 treatment enhanced the sphere-formation capability of U87 TS cells and patient-derived glioma TS cells (G027, Fig. 1g, h). Furthermore, GDF15 significantly increased the proportion of 5-ethynyl-20-deoxyuridine (EdU)-incorporated cells, indicating the promotion of cell division in GSCLCs (Fig. 1i, j). Upon blocking LIF signaling with a neutralizing antibody, we observed that the GSC marker expression, sphere formation, and cell division of GDF15-treated U87 TS cells were all repressed (Fig. 1k–m). Taken together, these data indicate that GDF15 can mediate the stem cell-like states of GSCLCs by activating LIF–STAT3 signaling.

### GDF15 stimulates LIF expression through upregulating c-Fos

**Fig. 2:**
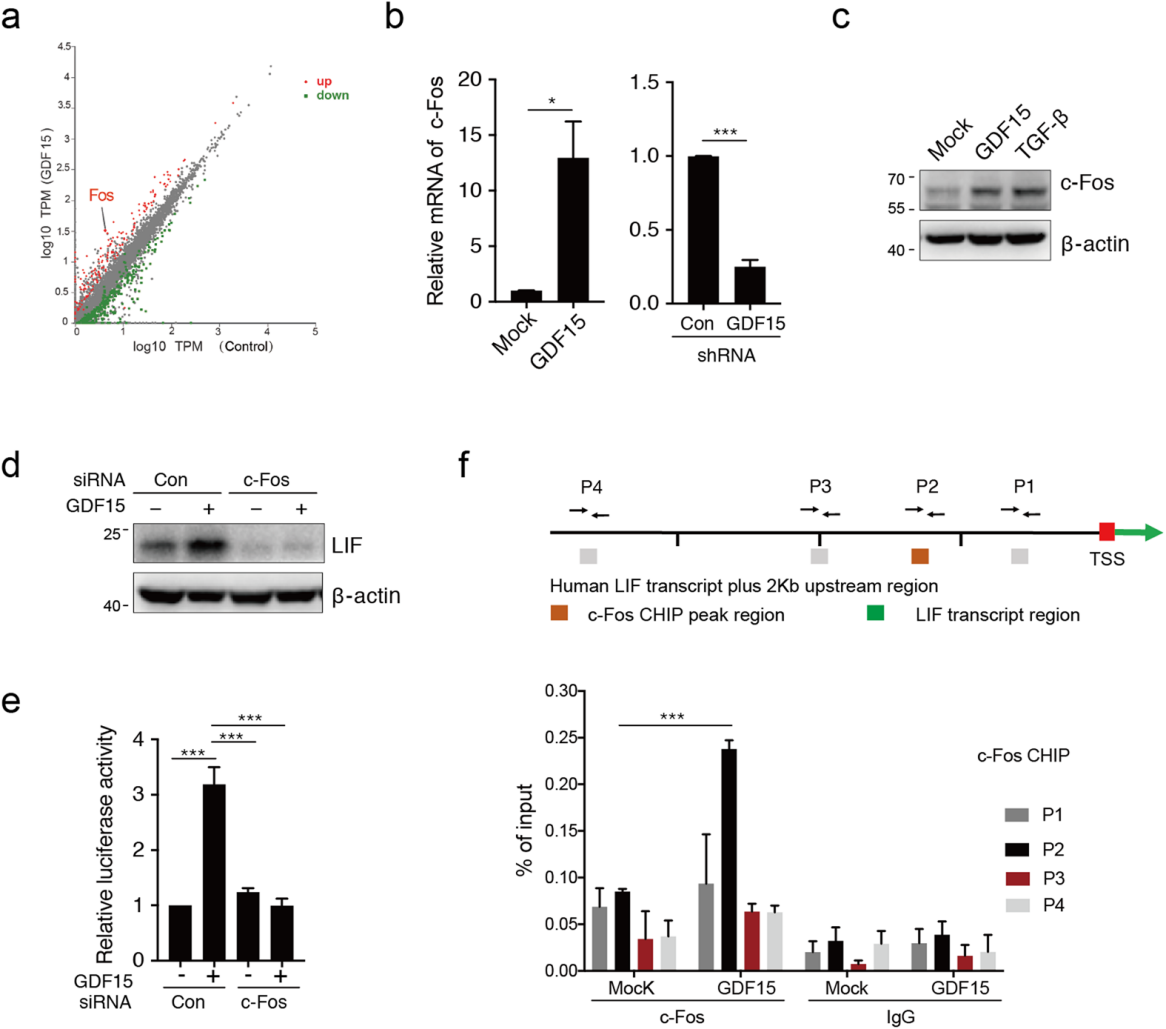
GDF15 upregulates LIF transcription via c-Fos binding to the promoter. **a** Scatter plot showing differentially expressed genes between control and 10 ng/ml GDF15-treated U87 TS cells. **b** qRT-PCR analysis of c-fos gene expression in U87 TS cells treated with GDF15 or short hairpin RNA lentivirus targeting the GDF15 gene. **c** Immunoblotting analysis of c-Fos and β-actin expression in U87 TS cells after treatment with GDF15 and TGF-β for 5 days. **d** Immunoblotting analysis of LIF protein expression in U87 TS cells after treatment with GDF15 and c-Fos siRNA or control siRNA. **e** U87 TS cells were transfected with a luciferase construct containing the LIF promoter and treated with GDF15 and c-Fos siRNA or control siRNA for 48 h, and luciferase activity was determined using a dual-luciferase reporter assay system. **f** Cells were incubated without or with GDF15 and subjected to ChIP assays using c-Fos or IgG isotype control antibodies. Bar chart representing the qPCR results for the immunoprecipitated LIF promoter. Values in **b**, **e**, and **f** are from three independent experiments and are expressed as mean ± s.e.m. *P < 0.05, ***P < 0.0005. Unpaired *t* test (**b**, **f**); one-way ANOVA with Tukey’s multiple comparison test (**e**).

To further analyze how GDF15 promotes LIF expression in GSCLCs, gene expression profiling was performed to identify the molecular changes triggered by GDF15 treatment. Compared with the negative control, treated U87 TS cells expressed higher levels of the transcription factor, c-Fos, whereas, knockdown of GDF15 in GSCLCs resulted in decreased expression of c-Fos (Fig. 2a–c). Moreover, the silencing of c-Fos reverted the induction of LIF expression by GDF15 (Fig. 2d and Supplementary Fig. 2a). Promoter activity analysis demonstrated that c-Fos knockdown reduced LIF promoter activation in response to GDF15 in GSCLCs (Fig. 2e), suggesting that GDF15 can activate the LIF promoter via the transcription factor c-Fos. To identify the crosstalk between c-Fos and the LIF promoter in a GDF15 context, we performed a ChIP assay in U87 TS cells in the presence or absence of GDF15. In GDF15-treated GSCLCs, c-Fos was only bound to the region (−792/−685) of the LIF promoter but not to the proximal region (−398/−269) or other two distal regions localized 1–2 kb upstream of the transcription start site (Fig. 2f). Collectively, these data indicate that GDF15 transcriptionally upregulates LIF by promoting the binding of c-Fos to the LIF promoter.

### Imiquimod upregulates the GDF15–LIF signaling and enhances the GSC-like phenotype in GSCLCs

**Fig. 3:**
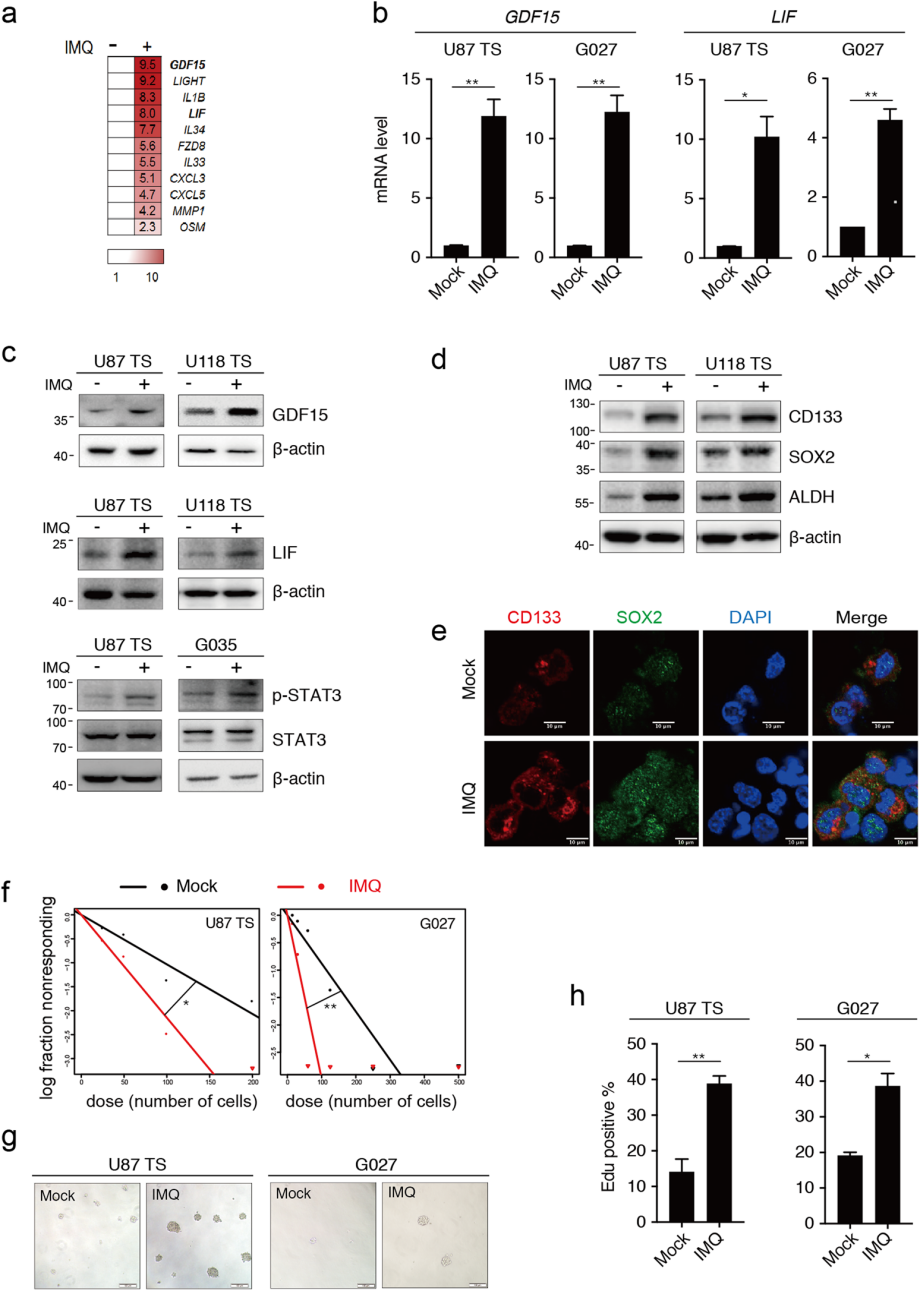
Imiquimod enhances the GSC-like phenotype. **a** The upregulation of genes identified by a transcriptomic analysis was confirmed by qRT-PCR in U87 TS treated with 5 μg/ml imiquimod (IMQ) for 5 days; data were normalized relative to the untreated groups. The scale reflects the levels of relative gene expression. **b** qRT-PCR analysis of GDF15 and LIF expression in U87 TS and G027 cells treated with IMQ. **c** Immunoblotting analysis of GDF15 and LIF protein expression in U87 TS and U118 TS treated with IMQ, as well as STAT3 and p-STAT3 levels in U87 TS and G035 treated with IMQ. **d** Immunoblotting analysis of CD133, SOX2, and ALDHA1 (ALDH) expression in U87 TS and U118 TS cells after treatment with IMQ for 5 days. **e** Immunocytochemical analysis with anti-CD133 and anti-SOX2 in U87 TS cells treated with IMQ for 5 days. Nuclei were counterstained with DAPI. Scale bar = 10 μm. **f** Effects of IMQ on the sphere-forming capacity of U87 TS and G027 cells as determined by extreme limiting dilution assay (*n* = 12). *P < 0.05, **P < 0.005; likelihood ratio test. **g** Representative images of U87 TS and G027 cells after treatment with 5 μg/ml IMQ for 7 and 10 days, respectively. Scale bar = 100 μm. **h** Cell division as determined by the EdU incorporation assay in U87 TS and G027 cells treated with IMQ. Values in **b** and **h** represent mean ± s.e.m. *P < 0.05, **P < 0.005 with unpaired *t* test.

Imiquimod (IMQ) was reported to have anti-tumor effects in several tumors via the inhibition of proliferation and induction of cell apoptosis^28–30^, but to be sparsely beneficial in glioblastoma (GBM). To investigate the impact of IMQ on the stemness of GSCs, we performed a transcriptomic analysis of GSCLCs (U87 TS) with or without IMQ treatment. Among the 23 upregulated genes, GDF15 and LIF were significantly upregulated after treatment with IMQ (Fig. 3a). Hence, we hypothesized that IMQ treatment could upregulate the GDF15–LIF pathway to promote the stemness properties of GSCs. In agreement, treatment with IMQ increased the protein and mRNA expression levels of GDF15 and LIF, as well as p-STAT3 (Fig. 3b, c).

Further, GSC markers (CD133, SOX2, and ALDH1A1) were upregulated in IMQ-treated GSCLCs (Fig. 3d, e). Also, after treatment with IMQ, GSCLCs exhibited a larger size and greater number of TS compared to the negative control (Fig. 3g). Similar results were also observed in the extreme limiting dilution assay. IMQ treatment significantly enhancing the sphere-forming ability in U87 TS and G027 cells (Fig. 3f). In addition, the proportion of EdU-incorporating cells was significantly increased by treatment with IMQ, indicating enhanced proliferation of U87 TS and G027 cells (Fig. 3h). Taken together, these results suggest a role for IMQ in promoting the GSC-like phenotype.

### GDF15–LIF pathway mediates the promotion of GSC-like phenotype by IMQ

**Fig. 4:**
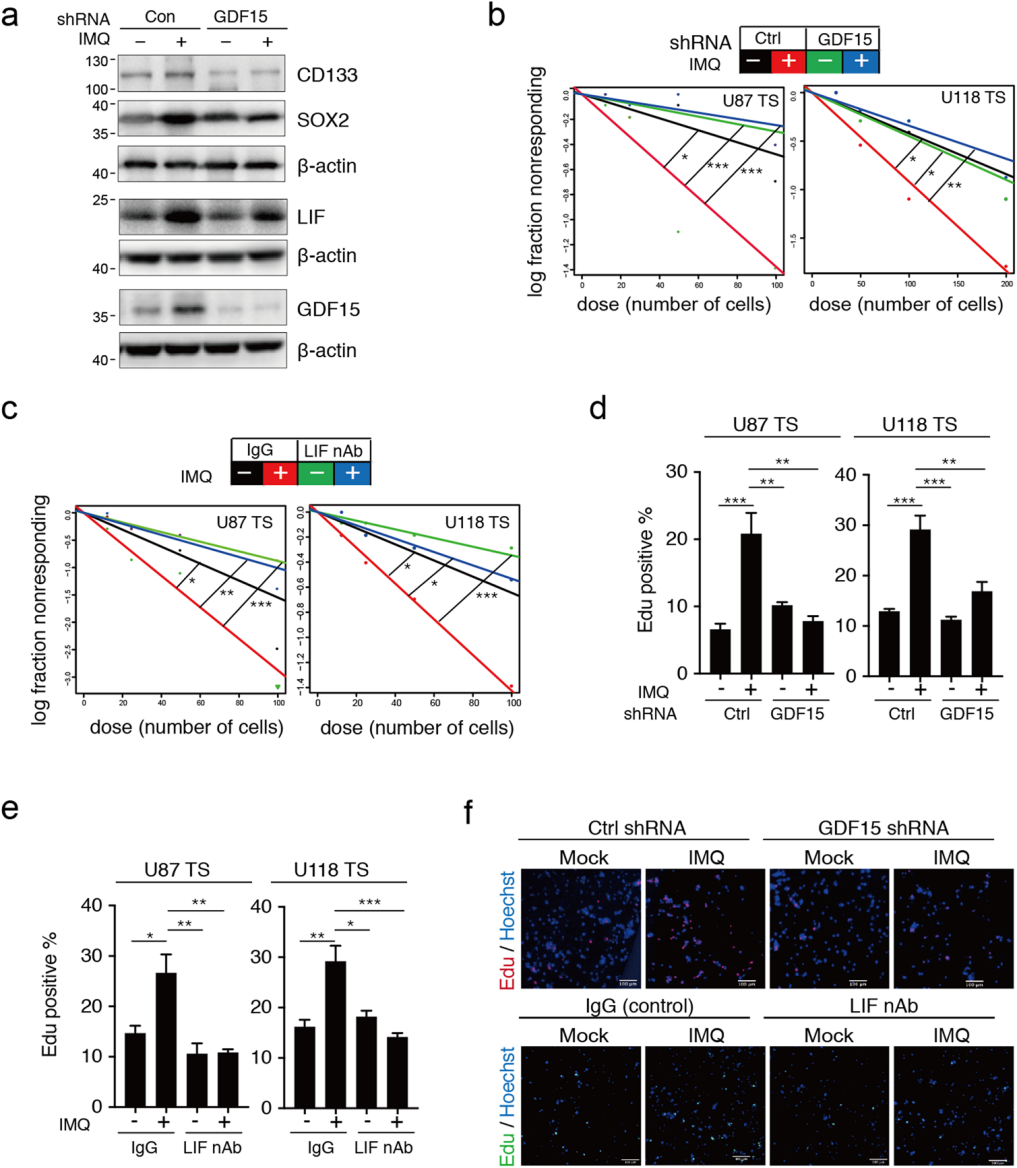
GDF15–LIF signaling mediates the GSC-like phenotype of GSC-like cells treated with Imiquimod. **a** U87 TS cells were treated with imiquimod (IMQ) for 5 days after short hairpin RNA lentivirus-mediated knockdown of GDF15, and the expression levels of CD133, SOX2, LIF, GDF15, and β-actin were determined by immunoblotting. **b**, **c** Effect of GDF15 knockdown or LIF neutralization on the IMQ-mediated induction of sphere formation by U87 TS and U118 TS cells as determined using extreme limiting dilution assay (*n* = 12). *P < 0.05, **P < 0.005, ***P < 0.0005; likelihood ratio test. **d**, **e** Cell division was determined by EdU incorporation assay in IMQ-treated U87 TS and U118 TS cells with shRNA-mediated GDF15 or anti-LIF-neutralizing antibody. Values are mean ± s.e.m. *P < 0.05, **P < 0.005, ***P < 0.0005. One-way ANOVA with Tukey’s multiple comparison test. **f** Representative images of EdU/Hoechst staining in U87 TS cells treated with IMQ as well as shRNA-mediated GDF15 or anti-LIF-neutralizing antibody. Scale bar = 100 μm.

Next, we explored whether the GDF15–LIF pathway is required for the induction of an increased GSC-like phenotype by IMQ treatment. As shown in Fig. 4a, enhanced expression levels of GSC markers and LIF were detected after IMQ treatment, and these effects were diminished with GDF15 knockdown. Moreover, blockade of LIF using a neutralizing antibody or silencing of GDF15 expression inhibited the IMQ-induced promotion of self-renewal capacity and cell proliferation, respectively (Fig. 4b–f). Together, these results demonstrate that the IMQ-enhanced GSC-like phenotype is indeed mediated via the GDF15–LIF pathway.

**Fig. 5:**
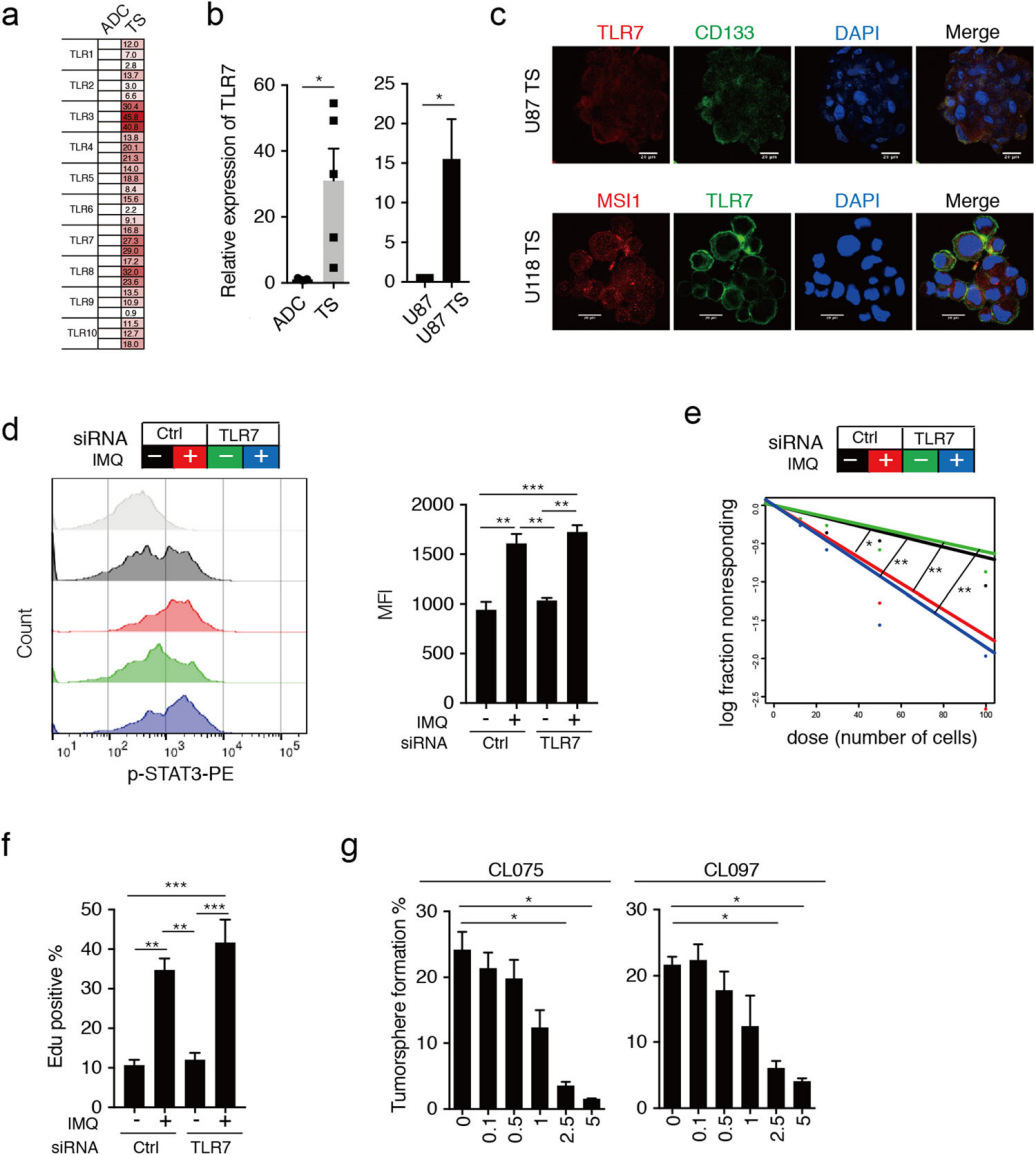
Knockdown of TLR7 does not prevent the promotion of GSC-like phenotype by imiquimod. **a** qRT-PCR analysis of the expression of human TLR mRNA in patient-derived TS relative to their adherent differentiated counterparts (ADCs); data were normalized to those for the ADCs. The scale reflects the level of relative gene expression (*n* = 3). **b** qRT-PCR analysis of TLR7 expression in patient-derived TS and ADCs (left, *n* = 5); qRT-PCR analysis of TLR7 expression in U87-MG and U87 TS cells (right). **c** Immunocytochemical analysis with anti-CD133, anti-TLR7, and anti-MSI1 in U87-MG and U118-MG cell-derived TS. Nuclei were counterstained with DAPI. Scale bar = 20 μm. **d** Flow cytometric analysis of the effect of TLR7 knockdown on p-STAT3 expression in G035 cells after imiquimod (IMQ) treatment. **e** Extreme limiting dilution assay to examine the effect of TLR7 knockdown on the induction of sphere formation by U87 TS cells after IMQ treatment (*n* = 12). *P < 0.05, **P < 0.005; likelihood ratio test. **f** The percentage of dividing U87 TS cells after treatment with IMQ as well as TLR7 knockdown as determined by EdU incorporation assay. **g** Bar chart representing the percentage of sphere-forming G035 cells after incubation with different concentrations of CL075 and CL097 for 14 days in the absence of growth factors. Values in **b**, **d**, **f**, and **g** represent mean ± s.e.m. *P < 0.05, **P < 0.005, ***P < 0.0005. Unpaired *t* test (**b**, **g**); one-way ANOVA with Tukey’s multiple comparison test (**d**, **f**).

IMQ is an established TLR7 agonist that can be used in tumor immunotherapy^31^. As shown in Fig. 5a–c, TLR7 expression was significantly higher in the patient- and glioma cell line-derived TS than their adherent differentiated counterparts (ADCs). To evaluate the involvement of TLR7 in the IMQ-enhanced GSC stemness properties, we transfected a validated siRNA targeting TLR7 mRNA into U87 TS cells (Supplementary Fig. 2b). Interestingly, TLR7 knockdown did not affect the p-STAT3 level, sphere formation, or cell division of GSCLCs in the presence of IMQ (Fig. 5d–f). We also tested the impact of two other TLR7 agonists on the regulation of stemness in GSCLCs. In contrast to our findings in IMQ-treated GSCLCs, treatment with CL075 and CL097 reduced sphere formation (Fig. 5g). Together, these results suggest that IMQ enhances the stemness properties of GSCs in a TLR7-independent manner.

### An ERK1/2 signaling pathway is critical for the promotion of GSC-like phenotype by GDF15

**Fig. 6:**
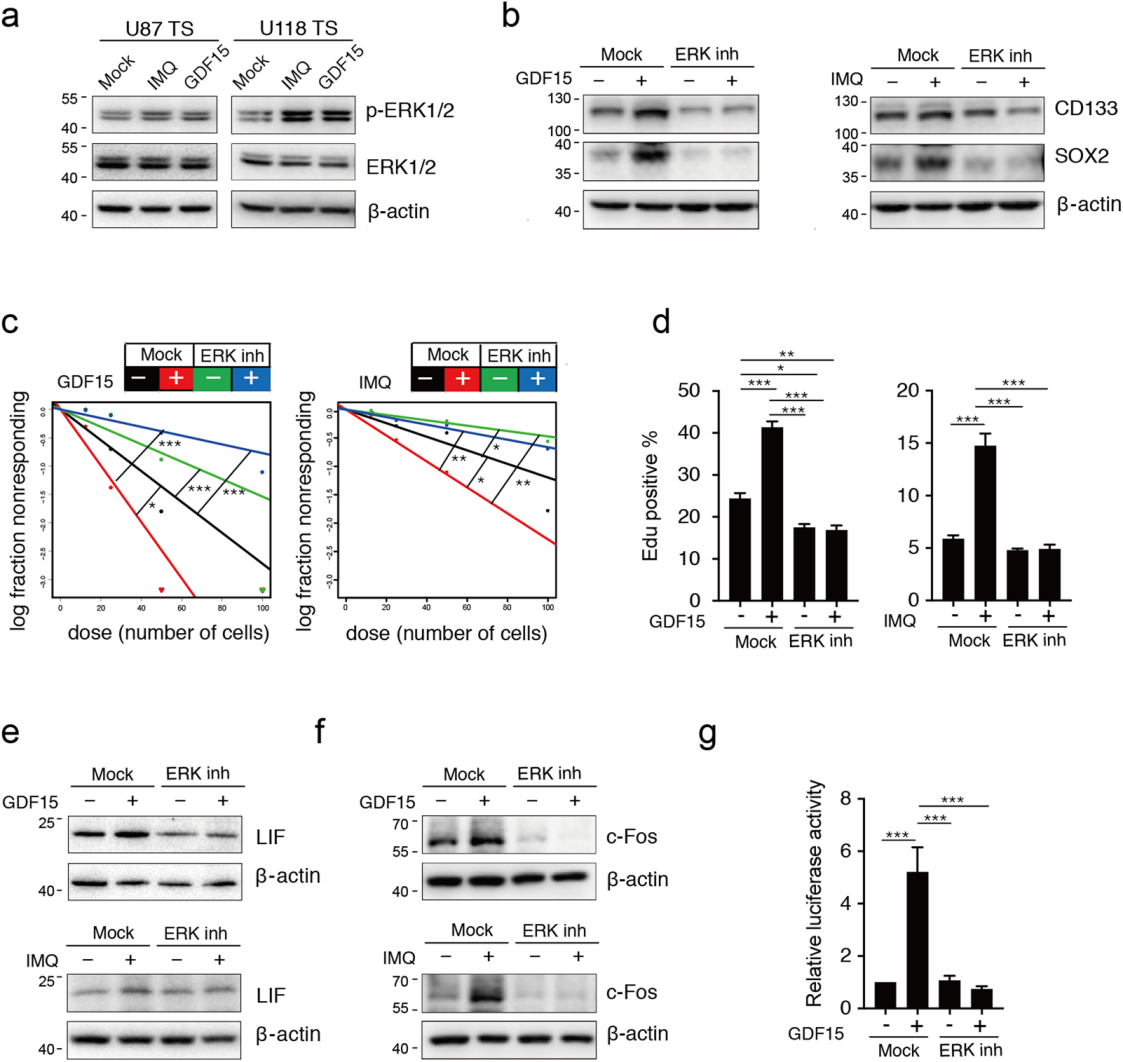
Induction of GSC-like phenotype by GDF15 is dependent on the ERK1/2 signaling pathway. **a** Immunoblotting analysis p-ERK1/2, ERK1/2, and β-actin expression in U87 TS and U118 TS cells after treatment with 5 μg/ml imiquimod (IMQ) and 10 ng/ml GDF15 for 5 days. **b** Immunoblotting analysis of CD133, SOX2, and β-actin expression in U87 TS cells after treatment with the ERK1/2 inhibitor PD98059 for 2 h and then with GDF15 and IMQ for 5 days. **c** Effects of ERK1/2 inhibitor on the induction of sphere formation by U87 TS cells in response to GDF15 and IMQ as determined by extreme limiting dilution assay (*n* = 12). *P < 0.05, **P < 0.005, ***P < 0.0005; likelihood ratio test. **d** Cell division among U87 TS cells treated with GDF15 and IMQ after PD98059 treatment, as determined by EdU incorporation assay. **e-f** Immunoblotting analysis of LIF, c-Fos, and β-actin expression in U87 TS cells treated with the ERK1/2 inhibitor PD98059 for 2 h and then GDF15 and IMQ for 5 days, respectively. **g** Luciferase activity detected in U87 TS cells transfected with a LIF luciferase reporter for 48 h following pretreatment with PD98059 for 2 h and then treatment with GDF15. Values in **d** and **g** represent mean ± s.e.m. *P < 0.05, **P < 0.005, ***P < 0.0005. One-way ANOVA with Tukey’s multiple comparison test.

TGF-β–Smads signaling can sustain GSC self-renewal and serve as the primary trigger to promote LIF transcription, while neither GDF15 nor IMQ induced the phosphorylation of Smads (Smad1/5/9, Smad2, and Smad3, Supplementary Fig. 3a). Previous research has confirmed that the MAPK signaling pathway is involved in the regulation of stemness^32,33^ and the induction of LIF expression^34^. Hence, we explored the modulating effect of MAPK signaling on the GDF15–LIF pathway in GSCs. Indeed, both IMQ and GDF15 induced rapid phosphorylation of ERK1/2 (Fig. 6a), without affecting the phosphorylation levels of p38 and JNK (Supplementary Fig. 3b). These observations imply that GDF15 functions upstream of ERK1/2 signaling in GSCs.

To evaluate whether the role of GDF15 in GSCs is dependent on ERK1/2 signaling, we used a selective MAPK kinase inhibitor PD98059 to block ERK1/2 activation. Following pretreatment with PD98059, GDF15- or IMQ-treated GSCLCs showed visible reductions in GSC marker expression, sphere formation, and proliferation (Fig. 6b–d). Further, PD98059 blocked the promotion of LIF expression in GDF15-treated as well as IMQ-treated GSCLCs (Fig. 6e). Similarly, elevated c-Fos expression and LIF promoter activity in the presence of GDF15 were also suppressed by blocking ERK1/2 signaling (Fig. 6f, g). These findings suggest that GDF15 mediates LIF expression and the GSC-like phenotype via the activation of ERK1/2 signaling.

## Discussion

In this study, we showed that GDF15 promotes the GSC-like phenotype in GSCLCs through activation of the LIF–STAT3 pathway. We first observed that GDF15 significantly upregulated LIF expression and STAT3 phosphorylation, which has been intimately associated with stemness-promotion in GSCs. Mechanistically, GDF15 increased the level of the transcription factor c-Fos and its binding to the LIF promoter, thus improving LIF transcription. Further, we demonstrated that the activation of the ERK1/2 signaling pathway is a critical step in the modulation of LIF transcription by GDF15, and treatment with an ERK1/2 inhibitor attenuated the GSC-like phenotype in GDF15-treated GSCLCs. Additionally, we showed here that the small-molecule drug IMQ facilitated the GSC-like phenotype by upregulating the GDF15–LIF pathway. Overall, our results suggest that GDF15 is an important pro-stemness factor in GBM.

Members of the TGF-β family are reported to promote the stemness of GSCs^23^. Here, we observed that the synthetic GDF15 protein increased the expression of GSC markers and the sphere-forming ability of GSCLCs, indicating that upregulated GDF15 expression correlates with increased stemness properties among GSCs. These results are consistent with previous research on the functions of GDF15 in breast cancer^19^ and multiple myeloma^35^. Previous work demonstrated the importance of LIF in maintaining GSC stemness^4,23,24^. LIF transcription is upregulated by TGF-β, thereby promoting the self-renewal of GSCs^23^. Our results indicate that GDF15 acts as an upstream regulatory factor for LIF transcription and promotes the GSC-like phenotype through activation of the LIF–STAT3 pathway. GSCs are associated with glioma recurrence^9,36^, and therapies targeting GSC demonstrated a marked growth inhibition and prolongation of animal survival^37,38^. Thus, GDF15 signaling may be required for the stemness properties of GSCs and maybe a critical therapeutic target for glioma treatment.

Various signaling pathways are involved in the regulation of the physiological functions of GSCs, and TGF-β-induced LIF transcription promotes the self-renewal of GSCs in a Smad2-dependent manner^23^. Therefore, we primarily examined the effects of Smads signaling on GSC stemness properties in the presence of GDF15; however, our results showed that Smads were not activated by GDF15 treatment. A significant correlation was also observed between the ERK1/2 signaling pathway and GSC stemness^33^. Both GDF15 and IMQ can activate the ERK1/2 signaling pathway^39–42^, which is also involved in the transcription of LIF^43,44^. Here, we showed that ERK1/2 activation might be a critical upstream event in the induction of LIF expression by GDF15. Notably, the self-renewal and proliferation of GSCLCs were promoted by GDF15 or IMQ treatment in an ERK1/2-dependent manner. In addition, GDF15 or IMQ treatment did not activate the JNK and P38 MAPK signaling pathways in GSCLCs (Supplementary Fig. 3b), though these signaling pathways are also suggested to be involved in cancer stemness^45,46^. Nevertheless, GSC stemness is regulated by complex signaling pathways, and it cannot be excluded that GDF15 could promote the stemness of GSCs through other potential signaling pathways.

IMQ is a small-molecule immunomodulator that can induce the secretion of interferons and pro-inflammatory cytokines by immune cells and facilitate the maturation of dendritic cells^47–49^. Previous studies demonstrated that IMQ can improve the efficacy of cancer immunotherapy and might serve as an effective adjuvant to dendritic cell-based tumor vaccines^31,50,51^. Furthermore, IMQ administration was found to have an anti-tumor effect in several epithelial tumors through the inhibition of proliferation and promotion of apoptosis^28–30^. In glioma, it has been reported that IMQ directly inhibits the proliferation of GL261 cells in a TLR7-independent manner^51^; however, the impact of IMQ on glioma cells and GSCs is incompletely understood. In this study, we identified a novel role for IMQ in enhancing the stemness properties of GSCLCs, indicating that certain immunomodulators may exert different pharmacological functions upon different cell types in the tumor microenvironment. Although outside the scope of the current paper, we also observed that IMQ inhibited the proliferation of glioma cells (U87-MG and U025) via the accumulation of cells in the G1 phase (Supplementary Fig. 4a–c), and almost none of those cells underwent apoptosis (Supplementary Fig. 4d). These results suggest that IMQ may promote the quiescent slow-cycling properties of glioma cells, which is a characteristic feature of cancer stem cells. In follow-up studies of glioma cells treated with IMQ, we will examine the cellular functions and the complex regulatory network.

In conclusion, the results obtained in this study reveal the ability of GDF15 to promote the GSC-like phenotype, at least in part through the induction of ERK1/2–c-Fos–LIF pathway signaling. Our findings highlight the critical role of GDF15 in regulating cancer cell stemness and provide a rationale for considering GDF15 as a potential target for the treatment of glioma.

## Materials and methods

### Cell lines and cell cultures

GBM samples were obtained from six glioma patients who underwent GBM resection between October 2014 and June 2016 in the Department of Neurosurgery of The First Hospital of Jilin University (Supplementary Table 1). Resected GBM samples were obtained during surgery and promptly dissociated to produce patient-derived glioma cells and spheroids, as described previously^23^. The design of this study was revised and approved by the ethics committee of the First Hospital of Jilin University (licenses 2014–28). All six patients provided informed written consent for participation in this study.

The U87-MG and U118-MG glioma cell lines were purchased from the American Type Culture Collection (Manassas, VA, USA), and cell line authentication was performed by short tandem repeat (STR) profiling. Cells were cultured in Dulbecco’s Modified Eagle’s Medium (DMEM, Hyclone, Logan, UT, USA) supplemented with 10% fetal bovine serum (FBS; Biological Industries, Kibbutz Beit Haemek, Israel) as well as 100 U/mL penicillin (Hyclone) and 100 μg/mL streptomycin sulfate (Hyclone). To establish GSCLCs, U87-MG and U118-MG cells were cultured in a chemically defined serum-free medium that included a DMEM/F12 base (Thermo Fisher Scientific, Waltham, MA, USA), 1× N2 (Thermo Fisher Scientific), 1× B27 (Thermo Fisher Scientific), 20 ng/ml epidermal growth factor (EGF, PeproTech, Rocky Hill, NJ, USA), 20 ng/ml basic fibroblast growth factor (bFGF, PeproTech), and 2.5 mg/ml heparin (Life Technologies, Grand Island, NY, USA).

### Reagents and antibodies

Recombinant human GDF15 (R&D Systems, Minneapolis, MN, USA), imiquimod (IMQ, InvivoGen, San Diego, CA, USA), neutralizing antibody against LIF (R&D Systems), and ERK1/2 inhibitor PD98059 (Cell Signaling Technology, Danvers, MA, USA) were used in this study. The antibodies used were as follows: anti-CD133/2 (Miltenyi Biotec, Auburn, CA, USA), anti-PROM1 (Sigma Aldrich, St. Louis, MO, USA), anti-Nestin (Merck-Millipore, Billerica, MA, USA), anti-Toll-like receptor 7 (anti-TLR7, Novus Biological Inc., Littleton, CO, USA), anti-Musashi (Merck-Millipore), anti-phospho-STAT3 (p-Tyr705, Cell Signaling Technology), anti-STAT3 (Thermo Fisher Scientific), anti-SOX2 (R&D Systems), anti-ALDH1 (Thermo Fisher Scientific), anti-β-actin (TransGen Biotech, Beijing, China), anti-GDF15 (Thermo Fisher Scientific), anti-LIF (Merck-Millipore), and anti-Fos (GeneTex, Irvine, CA, USA). The Smad2/3 Antibody Sampler Kit (Cell Signaling Technology) and Smad1/5/9 Antibody Sampler Kit (Cell Signaling Technology) were also employed.

### Quantitative real-time PCR

The total RNA was extracted from GSCLCs using the EasyPure RNA Kit (Transgen Biotech) following the manufacturer’s protocol, and the cDNA was synthesized using a TransScript Reverse Transcriptase kit (Transgen Biotech). Next, quantitative reverse transcription-polymerase chain reaction (RT-PCR) was performed using FastStart DNA Master SYBR Green (Roche Diagnostics, Indianapolis, IN, USA) on an ABI Step One RT-PCR system (Applied Biosystems, Foster City, CA, USA). Gene-specific primers were synthesized by GENEWIZ (Suzhou, China; Supplementary Table 2). The relative expression levels of TLRs, GDF15, LIF, and c-Fos were normalized to β-actin expression level and determined using the 2^−ΔΔCt^ method.

### Flow cytometry

Tumorspheres were dissociated into a single-cell suspension and labeled with anti-phospho-STAT3 antibody (Cell Signaling Technology). Next, treated cells underwent flow cytometric analysis on a BD LSR Fortessa (BD Biosciences, Franklin Lakes, NJ, USA), and the obtained data were analyzed using FlowJo software (BD Biosciences).

### Cell proliferation assay

The impact of GDF15 on cell proliferation was analyzed with a Cell-Light EdU In Vitro Kit (RiboBio Co., Guangzhou, China). Briefly, GSCLCs were cultured in six-well culture plates in a medium containing the different stimuli for 4 days. Next, cells were harvested, stained following the manufacturer’s protocol, and visualized on an inverted fluorescence microscope (IX71, Olympus, Tokyo, Japan). Images were analyzed using ImageJ software (National Institutes of Health, Bethesda, MD, USA).

### Extreme limiting dilution assay

TS were collected and dissociated into single-cell suspensions before plating into 96-well plates in 200 µl serum-free medium/well (~12.5–200 cells/well). Next, the percentage of wells containing spheres was analyzed after 10–14 days by extreme limiting dilution analysis (bioinf.wehi.edu.au/software/elda/)^52^.

### Cell lysis and immunoblotting

Total proteins were extracted from the treated GSCLCs with radioimmunoprecipitation assay (RIPA) buffer (Cell Signaling Technology) supplemented with protease/phosphatase inhibitors (MCE, Monmouth Junction, NJ, USA). Then the protein concentration in each sample was detected by bicinchoninic acid (BCA) protein assay according to the standard protocol. The denatured proteins were separated by sodium dodecyl sulfide (SDS)-polyacrylamide gel electrophoresis (PAGE) and transferred onto polyvinylidene difluoride (PVDF) membranes (Merck-Millipore). After blocking, the membranes were incubated with the primary antibodies at 4 °C overnight and then with horseradish peroxidase-conjugated secondary antibodies for 1 h at room temperature. Blots were visualized using Enhanced Chemiluminescence (ECL) Plus detection reagent (PerkinElmer, Boston, MA, USA).

### RNA interference

Lentiviral vectors and the concentrated lentivirus were purchased from Vigene Biosciences, Inc. (Shandong, China). Lentiviral vectors expressing three shRNA constructs targeting GDF15 (shGDF15 1#, shGDF15 2#, and shGDF15 3#, Supplementary table 3) were used to silence GDF15 expression. Small interfering RNAs (siRNAs) targeting TLR7, c-Fos, and control siRNA (all purchased from Santa Cruz Biotechnology, Santa Cruz, CA, USA) were introduced into GSCLCs with jetPEI™ transfection reagent (Polyplus-transfection, Illkirch, France) following the manufacturers’ protocols.

### Chromatin immunoprecipitation (ChIP) assay

A ChIP assay kit (Merck-Millipore) was used to explore the binding of c-Fos to the LIF promoter site according to the manufacturer’s protocol. Following reverse crosslinking, the DNA was treated with proteinase K and purified using a PCR purification kit (Transgen Biotech). Next, the purified DNA was subjected to qRT-PCR (Supplementary Table 4).

### Promoter luciferase assay

LIF luciferase reporter plasmids were obtained from OBiO Technology (Shanghai, China). For reporter constructs, the 1326-bp promoter region (70/+1255) of the LIF gene was cloned into the pGL4.1 firefly luciferase reporter vector. phRL-TK containing the Renilla luciferase gene was used as an internal standard for transfection efficiency. Cells were seeded into 24-well plates at a density of 1 × 10^5^ cells/well and co-transfected with 500 ng luciferase construct plasmid or an empty reporter vector DNA and 0.05 μg phRL-TK using jetPEI™ transfection reagent for 48 h (Polyplus-transfection). The cells were then lysed, and the luciferase activity was examined with the dual-luciferase reporter assay kit (Transgen Biotech) on a microplate reader with a multi-wavelength measurement system (Synergy™ H1, BioTek Instruments, Inc., Winooski, VT, USA). Luciferase activity was calculated relative to the Renilla luciferase activity.

### Statistical analysis

Data are presented as mean ± standard error of the mean (s.e.m.). Differences between different groups were analyzed by unpaired *t* test or one-way analysis of variance (ANOVA) followed by Tukey’s test. Statistical analysis was performed using GraphPad Prism (San Diego, CA, USA).

## Supplementary information

Supplementary Table 1

Supplementary Table 2

Supplementary Table 3

Supplementary Table 4

Supplementary Figure legends

Supplementary Figure 1

Supplementary Figure 2

Supplementary Figure 3

Supplementary Figure 4

## References

[1] Fuchs E, Tumbar T, Guasch G (2004). Socializing with the neighbors: stem cells and their niche. Cell.

[2] Annovazzi, L., Mellai, M. & Schiffer, D. Chemotherapeutic drugs: DNA damage and repair in glioblastoma. *Cancers* **9**, 57 (2017). 10.3390/cancers9060057 PMC5483876 28587121

[3] Bao S (2006). Glioma stem cells promote radioresistance by preferential activation of the DNA damage response. Nature.

[4] Singh SK (2003). Identification of a cancer stem cell in human brain tumors. Cancer Res.

[5] Hemmati HD (2003). Cancerous stem cells can arise from pediatric brain tumors. Proc. Natl Acad. Sci. USA.

[6] Rasper M (2010). Aldehyde dehydrogenase 1 positive glioblastoma cells show brain tumor stem cell capacity. Neuro. Oncol..

[7] Strojnik T, Rosland GV, Sakariassen PO, Kavalar R, Lah T (2007). Neural stem cell markers, nestin and Musashi proteins, in the progression of human glioma: correlation of nestin with prognosis of patient survival. Surg. Neurol..

[8] Tao Z (2018). Autophagy suppresses self-renewal ability and tumorigenicity of glioma-initiating cells and promotes Notch1 degradation. Cell Death Dis..

[9] Sorensen MD (2015). Chemoresistance and chemotherapy targeting stem-like cells in malignant glioma. Adv. Exp. Med. Biol..

[10] Paw I, Carpenter RC, Watabe K, Debinski W, Lo HW (2015). Mechanisms regulating glioma invasion. Cancer Lett..

[11] Cekanova M (2009). Nonsteroidal anti-inflammatory drug-activated gene-1 expression inhibits urethane-induced pulmonary tumorigenesis in transgenic mice. Cancer Prev. Res..

[12] Husaini Y (2012). Macrophage inhibitory cytokine-1 (MIC-1/GDF15) slows cancer development but increases metastases in TRAMP prostate cancer prone mice. PLoS ONE.

[13] Baek SJ (2006). Nonsteroidal anti-inflammatory drug-activated gene-1 over expression in transgenic mice suppresses intestinal neoplasia. Gastroenterology.

[14] Vanhara P, Hampl A, Kozubik A, Soucek K (2012). Growth/differentiation factor-15: prostate cancer suppressor or promoter?. Prostate Cancer Prostatic Dis..

[15] Li S, Ma YM, Zheng PS, Zhang P (2018). GDF15 promotes the proliferation of cervical cancer cells by phosphorylating AKT1 and Erk1/2 through the receptor ErbB2. J. Exp. Clin. Cancer Res..

[16] Roth P (2010). GDF-15 contributes to proliferation and immune escape of malignant gliomas. Clin. Cancer Res..

[17] Shnaper S (2009). Elevated levels of MIC-1/GDF15 in the cerebrospinal fluid of patients are associated with glioblastoma and worse outcome. Int. J. Cancer.

[18] Guo J (2016). S100A4 influences cancer stem cell-like properties of MGC803 gastric cancer cells by regulating GDF15 expression. Int. J. Oncol..

[19] Sasahara A (2017). An autocrine/paracrine circuit of growth differentiation factor (GDF) 15 has a role for maintenance of breast cancer stem-like cells. Oncotarget.

[20] Xu Q (2017). Growth differentiation factor 15 induces growth and metastasis of human liver cancer stem-like cells via AKT/GSK-3beta/beta-catenin signaling. Oncotarget.

[21] Kim KH (2016). NSAID-activated gene 1 mediates pro-inflammatory signaling activation and paclitaxel chemoresistance in type I human epithelial ovarian cancer stem-like cells. Oncotarget.

[22] Bauer S, Patterson PH (2006). Leukemia inhibitory factor promotes neural stem cell self-renewal in the adult brain. J. Neurosci..

[23] Penuelas S (2009). TGF-beta increases glioma-initiating cell self-renewal through the induction of LIF in human glioblastoma. Cancer Cell.

[24] Edwards LA (2017). ZEB1 regulates glioma stemness through LIF repression. Sci. Rep..

[25] Beier D (2007). CD133(+) and CD133(-) glioblastoma-derived cancer stem cells show differential growth characteristics and molecular profiles. Cancer Res..

[26] Ernst M, Jenkins BJ (2004). Acquiring signalling specificity from the cytokine receptor gp130. Trends Genet.

[27] McLean K (2018). Leukemia inhibitory factor functions in parallel with interleukin-6 to promote ovarian cancer growth. Oncogene.

[28] Han JH (2013). In vitro and in vivo growth inhibition of prostate cancer by the small molecule imiquimod. Int. J. Oncol..

[29] Schon M (2003). Tumor-selective induction of apoptosis and the small-molecule immune response modifier imiquimod. J. Natl Cancer Inst..

[30] Schon MP (2004). Death receptor-independent apoptosis in malignant melanoma induced by the small-molecule immune response modifier imiquimod. J. Investig. Dermatol..

[31] Vasilakos JP, Tomai MA (2013). The use of Toll-like receptor 7/8 agonists as vaccine adjuvants. Expert Rev. Vaccines.

[32] Han Y (2016). Downregulation of lncRNA-MALAT1 affects proliferation and the expression of stemness markers in glioma stem cell line SHG139S. Cell Mol. Neurobiol..

[33] Nakada M (2013). Integrin alpha3 is overexpressed in glioma stem-like cells and promotes invasion. Br. J. Cancer.

[34] Jia C, Keasey MP, Lovins C, Hagg T (2018). Inhibition of astrocyte FAK-JNK signaling promotes subventricular zone neurogenesis through CNTF. Glia.

[35] Tanno T (2014). Growth differentiating factor 15 enhances the tumor-initiating and self-renewal potential of multiple myeloma cells. Blood.

[36] Jhaveri N, Chen TC, Hofman FM (2016). Tumor vasculature and glioma stem cells: contributions to glioma progression. Cancer Lett..

[37] Zhu Z (2014). Targeting self-renewal in high-grade brain tumors leads to loss of brain tumor stem cells and prolonged survival. Cell Stem Cell.

[38] Waldron NN (2011). Targeting tumor-initiating cancer cells with dCD133KDEL shows impressive tumor reductions in a xenotransplant model of human head and neck cancer. Mol. Cancer Ther..

[39] Huang X (2018). ERK inhibitor JSI287 alleviates imiquimod-induced mice skin lesions by ERK/IL-17 signaling pathway. Int. Immunopharmacol..

[40] Hassan F (2009). Involvement of interleukin-1 receptor-associated kinase (IRAK)-M in toll-like receptor (TLR) 7-mediated tolerance in RAW 264.7 macrophage-like cells. Cell Immunol..

[41] Griner SE, Joshi JP, Nahta R (2013). Growth differentiation factor 15 stimulates rapamycin-sensitive ovarian cancer cell growth and invasion. Biochem. Pharmacol..

[42] Jin YJ, Lee JH, Kim YM, Oh GT, Lee H (2012). Macrophage inhibitory cytokine-1 stimulates proliferation of human umbilical vein endothelial cells by up-regulating cyclins D1 and E through the PI3K/Akt-, ERK-, and JNK-dependent AP-1 and E2F activation signaling pathways. Cell Signal.

[43] Arthan D, Hong SK, Park JI (2010). Leukemia inhibitory factor can mediate Ras/Raf/MEK/ERK-induced growth inhibitory signaling in medullary thyroid cancer cells. Cancer Lett..

[44] Li Z (2012). BMP4 signaling acts via dual-specificity phosphatase 9 to control ERK activity in mouse embryonic stem cells. Cell Stem Cell.

[45] Sato A (2014). Pivotal role for ROS activation of p38 MAPK in the control of differentiation and tumor-initiating capacity of glioma-initiating cells. Stem Cell Res.

[46] Kitanaka C, Sato A, Okada M (2013). JNK signaling in the control of the tumor-initiating capacity associated with cancer stem cells. Genes Cancer.

[47] Lore K (2003). Toll-like receptor ligands modulate dendritic cells to augment cytomegalovirus- and HIV-1-specific T cell responses. J. Immunol..

[48] Hemmi H (2002). Small anti-viral compounds activate immune cells via the TLR7 MyD88-dependent signaling pathway. Nat. Immunol..

[49] Gibson SJ (2002). Plasmacytoid dendritic cells produce cytokines and mature in response to the TLR7 agonists, imiquimod and resiquimod. Cell Immunol..

[50] Adams S (2008). Immunization of malignant melanoma patients with full-length NY-ESO-1 protein using TLR7 agonist imiquimod as vaccine adjuvant. J. Immunol..

[51] Xiong Z, Ohlfest JR (2011). Topical imiquimod has therapeutic and immunomodulatory effects against intracranial tumors. J. Immunother..

[52] Hu Y, Smyth GK (2009). ELDA: extreme limiting dilution analysis for comparing depleted and enriched populations in stem cell and other assays. J. Immunol. Methods.

