# Supplementary material for: GDF15 promotes glioma stem cell-like phenotype via regulation of ERK1/2–c-Fos–LIF signaling
Source: Cell Death Discov. 2021 Jan 11;7:3. doi: 10.1038/s41420-020-00395-8 (PMC7801449; doi:10.1038/s41420-020-00395-8)
Supplement: Supplementary file 1 — Supplementary Table 1 [file 41420_2020_395_MOESM1_ESM.docx]

**Supplementary Table 1. Profiles of the patients**

| Sample # | Sphere cells # | Primary GBM cells# | Age  (years) | Sex | Diagnosis | WHO Grade |
| --- | --- | --- | --- | --- | --- | --- |
| Patient #12 | G012 | NA | 44 | M | GBM | IV |
| Patient #25 | G025 | U025 | 78 | M | GBM | IV |
| Patient #27 | G027 | U027 | 53 | F | GBM | IV |
| Patient #35 | G035 | NA | 67 | M | GBM | IV |
| Patient #38 | G038 | U038 | 58 | F | GBM | IV |
| Patient #40 | G040 | U040 | 66 | M | GBM | IV |

GBM, glioblastoma multiforme
