# Supplementary material for: GDF15 promotes glioma stem cell-like phenotype via regulation of ERK1/2–c-Fos–LIF signaling
Source: Cell Death Discov. 2021 Jan 11;7:3. doi: 10.1038/s41420-020-00395-8 (PMC7801449; doi:10.1038/s41420-020-00395-8)
Supplement: Supplementary file 2 — Supplementary Table 2 [file 41420_2020_395_MOESM2_ESM.docx]

**Supplementary Table 2. Primers used for quantitative real-time PCR**

| Gene | Sequence |
| --- | --- |
| TLR7 | F-AAAATGGTGTTTCCAATGTGG |
|  | R-GGCAGAGTTTTAGGAAACCATC |
| GDF 15 | F-GGGAAGATTCGAACACCGA |
|  | R-ACTTCTGGCGTGAGTATCC |
| LIF | F-GTCCAGGTTGTTGGGGAAC |
|  | R-TGCCAATGCCCTCTTTATTC |
| c-Fos | F-GCGTTGTGAAGACCATGAC |
|  | R-CTTCTTCTGGAGATAACTGTTCC |
| β-actin | F-TCGTGCGTGACATTAAGGAG |
|  | R-ATGCCAGGGTACATGGTGGT |
