# Supplementary material for: GDF15 promotes glioma stem cell-like phenotype via regulation of ERK1/2–c-Fos–LIF signaling
Source: Cell Death Discov. 2021 Jan 11;7:3. doi: 10.1038/s41420-020-00395-8 (PMC7801449; doi:10.1038/s41420-020-00395-8)
Supplement: Supplementary file 3 — Supplementary Table 3 [file 41420_2020_395_MOESM3_ESM.docx]

**Supplementary Table 3. Target sequences used to silence GDF15 expression**

| No. | Sequence |
| --- | --- |
| GDF15 1# | AACCTGCACAGCCATGCCCGG |
| GDF15 2# | AACTCAGGACGGTGAATGGCT |
| GDF15 3# | GGATACTCACGCCAGAAGTGCG |
