# Supplementary material for: GDF15 promotes glioma stem cell-like phenotype via regulation of ERK1/2–c-Fos–LIF signaling
Source: Cell Death Discov. 2021 Jan 11;7:3. doi: 10.1038/s41420-020-00395-8 (PMC7801449; doi:10.1038/s41420-020-00395-8)
Supplement: Supplementary file 4 — Supplementary Table 4 [file 41420_2020_395_MOESM4_ESM.docx]

**Supplementary Table 4. PCR primers used in chromatin immunoprecipitation analyses**

| No. | Sequences |
| --- | --- |
| P1 | F- GAAGCACCTTCCCAGAGTGT |
|  | R- CTGACCACTGTACCTGTGAG |
| P2 | F- TGCTAGTCCAGACTCGCTAC |
|  | R- CCGGGAGTTGTCTGAAGATG |
| P3 | F- GCAGCTGCAGAACTCTTGAG |
|  | R- ATGGACCCCAAATCCATGTG |
| P4 | F- ACACCTTGATCTCAGGTCAG |
|  | R- ATGCCGTCCCTAAAGCTGCC |
