# Supplementary material for: GDF15 promotes glioma stem cell-like phenotype via regulation of ERK1/2–c-Fos–LIF signaling
Source: Cell Death Discov. 2021 Jan 11;7:3. doi: 10.1038/s41420-020-00395-8 (PMC7801449; doi:10.1038/s41420-020-00395-8)
Supplement: Supplementary file 5 — Supplementary Figure legends [file 41420_2020_395_MOESM5_ESM.docx]

**Supplementary Figure legends**

**Supplementary Fig. 1 Characteristics of patient- and glioma cell line-derived tumorspheres.**

**a** Representative images of patient-derived tumorsphere (TS) G012, which were cultured in serum-free media with EGF and bFGF. Scale bar = 50 μm. **b** Representative images of glioma cell line-derived TS (U87 TS and U118 TS) cultured in serum-free media. Scale bar = 100 μm. **c** Representative images glioma cells named U025 (‘‘Differentiated’’) derived from G025 cells cultured in media containing 10% fetal bovine serum. Scale bar = 100 μm. **d** CD133, Nestin, SOX2, and GFAP expression patient-derived TS cells (G035 and G038) and differentiated cells (U025 and U027) were determined by flow cytometry. **e** Immunostaining of spheres. U87 TS and U118 TS were stained with CD133 and Nestin. Nuclei were counterstained with DAPI. Scale bar = 20 μm.

**Supplementary Fig. 2 Knockdown validation of RNA interfering molecules**

**a**-**b** C-Fos and TLR7 protein levels were analyzed in U87 TS cells treated with c-Fos siRNA, TLR7 siRNA, and control siRNA using immunoblotting.

**Supplementary Fig. 3 Imiquimod and GDF15 do not increase the phosphorylation of P38, JNK, and Smads**

**a-b** U87 TS cells were treated with 5 μg/ml imiquimod (IMQ) and 10 ng/ml GDF15 for 3 days, and the levels of p-P38, P38, p-JNK, JNK, p-Smads, Smads, and β-actin were determined by immunoblotting, respectively.

**Supplementary Fig. 4 Imiquimod treatment arrests glioma cells at the G1 phase, does not induce apoptosis.**

**a** Crystal violet staining on day 7 showing decreased proliferation in U025 and U040 cells with imiquimod (IMQ, 5 μg/ml) treatment compared to normal control. **b-c** U87-MG and U025 cells were incubated with different IMQ concentrations (0, 1, 2.5, 5, and 10 μg/ml) for 3 days, and cell cycle arrest was analyzed using PI staining as well as flow cytometry analyses. Values are mean ± s.e.m. *P < 0.05, **P < 0.005. One-way ANOVA with Tukey’s Multiple Comparison Test. **d** U87-MG and U118-MG cells were treated with various concentrations of IMQ for 48 h. The harvested cells were then stained with Annexin V/7-AAD, and flow cytometry was performed to analyze apoptotic rates.
