# Supplementary figures and images for: GDF15 promotes glioma stem cell-like phenotype via regulation of ERK1/2–c-Fos–LIF signaling
Source: Cell Death Discov. 2021 Jan 11;7:3. doi: 10.1038/s41420-020-00395-8 (PMC7801449; doi:10.1038/s41420-020-00395-8)

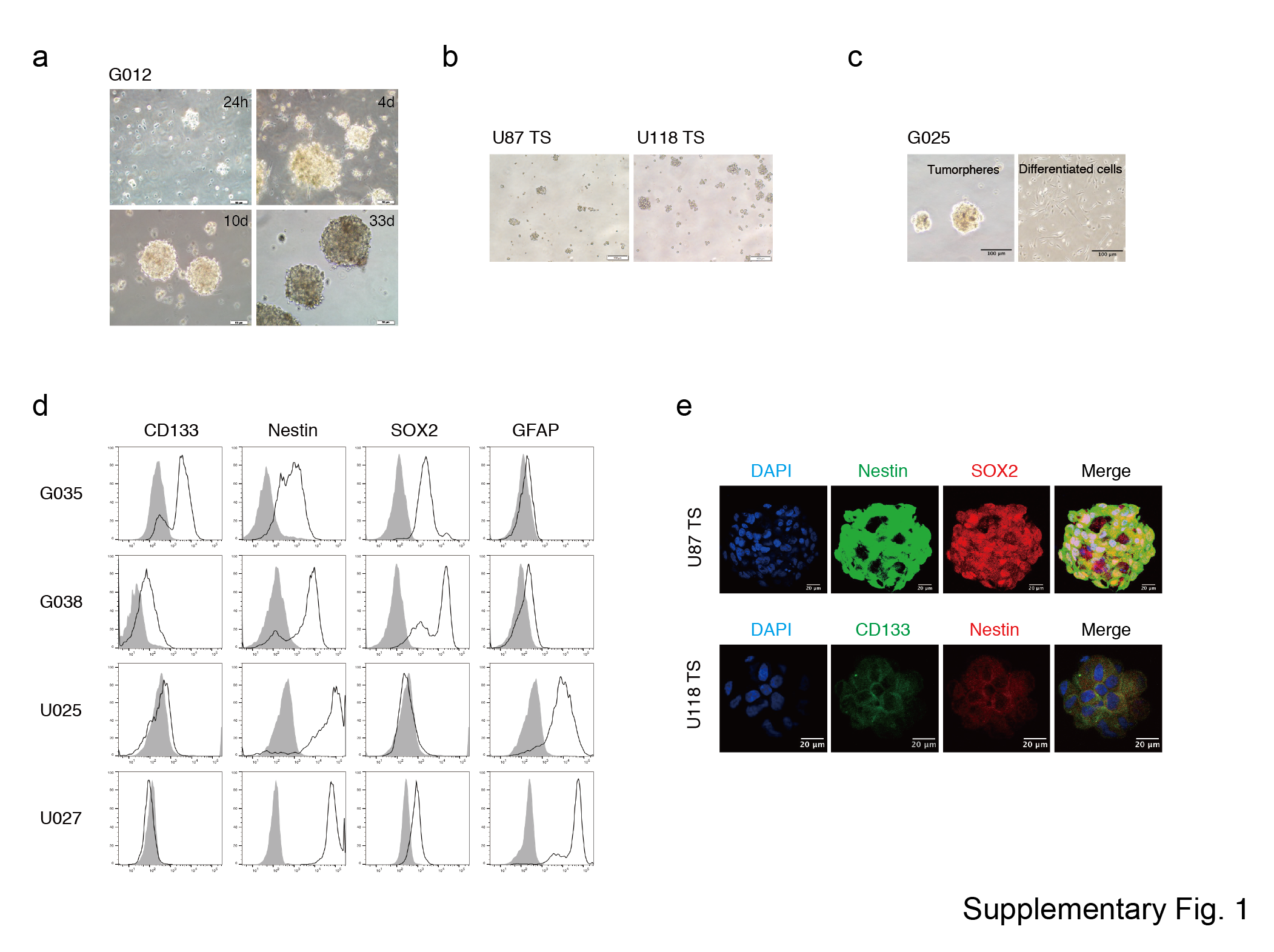

Supplement: Supplementary file 6 — Supplementary Figure 1 [file 41420_2020_395_MOESM6_ESM.png]

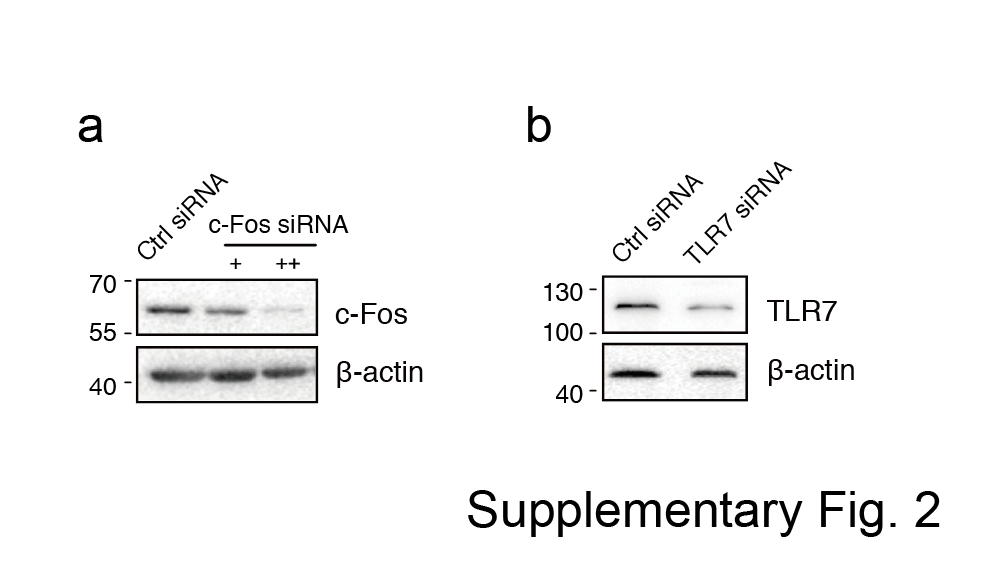

Supplement: Supplementary file 7 — Supplementary Figure 2 [file 41420_2020_395_MOESM7_ESM.png]

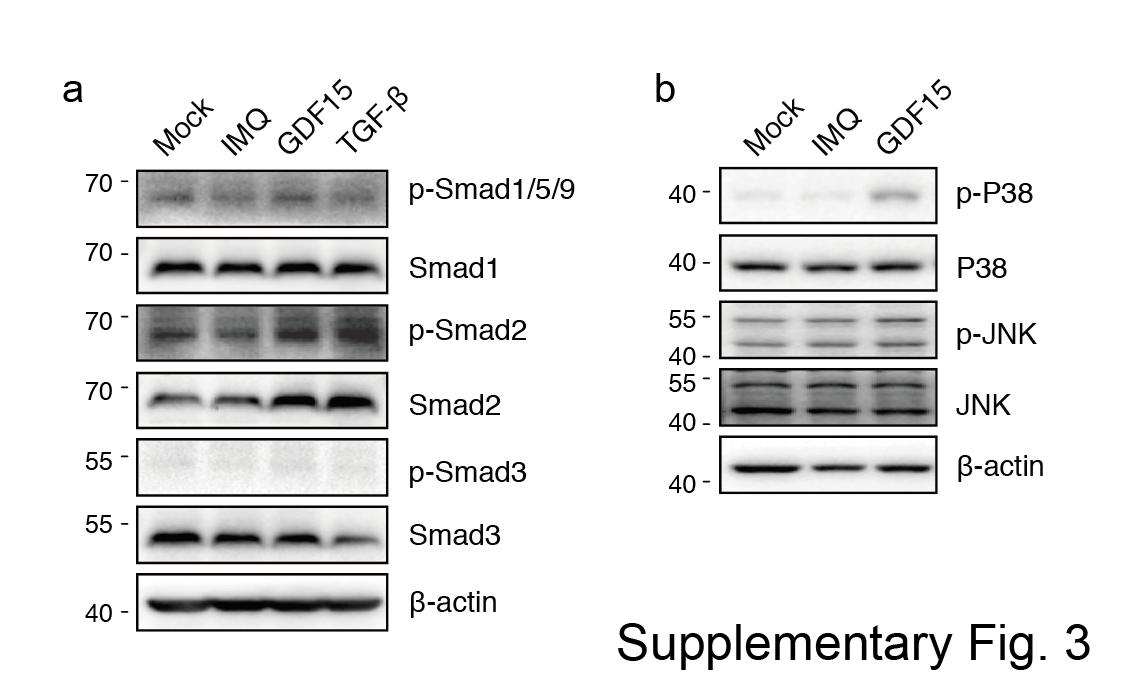

Supplement: Supplementary file 8 — Supplementary Figure 3 [file 41420_2020_395_MOESM8_ESM.png]

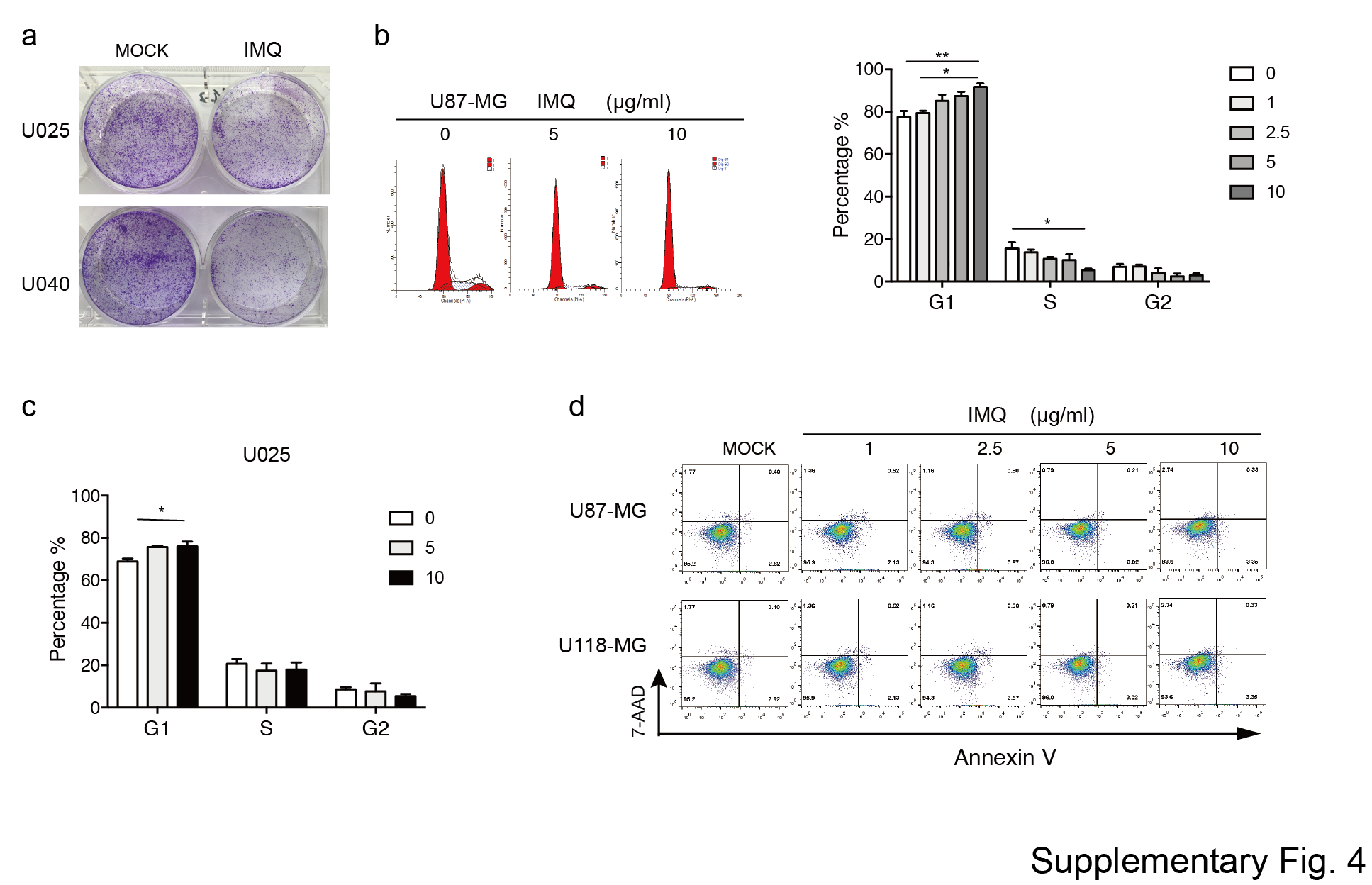

Supplement: Supplementary file 9 — Supplementary Figure 4 [file 41420_2020_395_MOESM9_ESM.png]
